# Entanglement as a resource to distinguish orthogonal product states

**DOI:** 10.1038/srep30493

**Published:** 2016-07-26

**Authors:** Zhi-Chao Zhang, Fei Gao, Tian-Qing Cao, Su-Juan Qin, Qiao-Yan Wen

**Affiliations:** 1 State Key Laboratory of Networking and Switching Technology, Beijing University of Posts and Telecommunications, Beijing, 100876, China; 2 State Key Laboratory of Cryptology, P.O. Box 5159, Beijing, 100878, China

## Abstract

It is known that there are many sets of orthogonal product states which cannot be distinguished perfectly by local operations and classical communication (LOCC). However, these discussions have left the following open question: What entanglement resources are necessary and/or sufficient for this task to be possible with LOCC? In *m* ⊗ *n*, certain classes of unextendible product bases (UPB) which can be distinguished perfectly using entanglement as a resource, had been presented in 2008. In this paper, we present protocols which use entanglement more efficiently than teleportation to distinguish some classes of orthogonal product states in *m* ⊗ *n*, which are not UPB. For the open question, our results offer rather general insight into why entanglement is useful for such tasks, and present a better understanding of the relationship between entanglement and nonlocality.

In quantum information theory, one of the main goals is to understand the power and limitation of quantum operations which can be implemented by local operations and classical communication (LOCC)^1^,^2^,^3^. For example, it is impossible, by LOCC, to transform a product state into an entangled state^4^, even with a nonzero probability. When global operators cannot be implemented by LOCC, it reflects the fundamental feature of quantum mechanics which is called nonlocality and has received wide attention in recent years^5^,^6^,^7^,^8^,^9^,^10^,^11^,^12^,^13^,^14^,^15^,^16^,^17^,^18^,^19^,^20^,^21^. A fundamental result in this area was the discovery by Bennett *et al.*^22^ of a complete orthogonal product basis which cannot be distinguished perfectly by LOCC. The authors dubbed this phenomenon “nonlocality without entanglement”, and various of related results have been presented^23^,^24^,^25^,^26^,^27^,^28^,^29^,^30^,^31^,^32^,^33^,^34^,^35^,^36^,^37^,^38^,^39^.

In fact, when the parties share enough entanglement, any set of locally indistinguishable states can always be distinguished by LOCC. That is, with enough entanglement, LOCC can be used to teleport^40^ the full multipartite state to a single party, and this party can then make a measurement to determine which state they were given. For example, in a bipartite quantum system *m* ⊗ *n*(2 ≤ *m* ≤ *n*), a maximally entangled state 
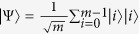
 is sufficient for distinguishing any set of orthogonal bipartite quantum states. This can be easily understood by imagining two spatially separated observers Alice and Bob: First, Alice uses the extra shared entanglement to teleport her qudit to Bob; then, this allows Bob, who now holds both qudits, to perform the desired measurement.

However, in recent years, entanglement has been shown to be a valuable resource^41^,^42^, allowing remote parties to communicate in ways which were previously not thought possible. Examples include the well-known protocols of teleportation^40^, dense coding^43^ and data hiding^44^,^45^. These results have caused a rapid growth in the new field of quantum information. In addition, people believe that discovering the potential power of quantum computers^46^ may also rely on entanglement. Thus, entanglement is a valuable resource and it also become important to ask if this task can be accomplished more efficiently.

In 2008, Cohen presented that certain classes of unextendible product bases (UPB) in *m* ⊗ *n*(*m* ≤ *n*) can be distinguished perfectly with a 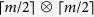 maximally entangled state^47^. A natural question to ask is whether this task can be completed more efficiently for the general locally indistinguishable orthogonal product states in *m* ⊗ *n*, which are not UPB.

In this paper, we will consider the locally indistinguishable orthogonal product states in the general bipartite quantum systems and present the LOCC protocols which, using entanglement as a resource, distinguish these states considerably more efficiently than teleportation. Specifically, in 5 ⊗ 5, we show a set of locally indistinguishable orthogonal product states can be distinguished by LOCC with a 2 ⊗ 2 maximally entangled state. Furthermore, for several classes of locally indistinguishable orthogonal product states on a higher-dimensional *m* ⊗ *n*, we present that only needing a 2 ⊗ 2 maximally entangled state, these states are also distinguishable by LOCC. Each of the locally indistinguishable orthogonal product states that we consider can be extended to a complete “nonlocality without entanglement” basis, and the latter can also be distinguished by the same (or slightly altered) protocols. Our results show that the locally indistinguishable quantum states may become distinguishable with a small amount of entanglement resources. And these results also present a better understanding of the relationship between entanglement and nonlocality.

## Results

### Local distinguishability of orthogonal product states in 5 ⊗ 5

In this section, we present that a set of locally indistinguishable orthogonal product states in 5 ⊗ 5, can be perfectly distinguished by LOCC with a 2 ⊗ 2 maximally entangled state. In 5 ⊗ 5, the following 21 orthogonal product states cannot be distinguished by LOCC^48^, and the structure of these states is different from ref. 48 only lying in exchange of Alice and Bob. For example, in ref. 48 |*ϕ*〉 = |0〉_*A*_|0 ± 1〉_*B*_, but in this paper |*ϕ*〉 = |0 ± 1〉_*A*_|0〉_*B*_.


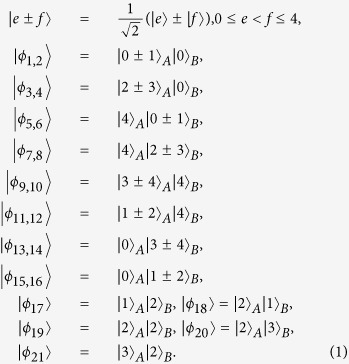


In the following, applying the proof method which was presented by Cohen^47^, we present a protocol which uses entanglement more efficiently than teleportation to distinguish these quantum states (1).

Theorem 1 *In* 5 ⊗ 5, a 2 ⊗ 2 *maximally entangled state is sufficient to perfectly distinguish the above 21 states by LOCC*.

*Proof.* First of all, Alice and Bob share a 2 ⊗ 2 maximally entangled state 
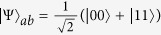
.

Then, Bob performs a two-outcome measurement, each outcome corresponding to a rank-5 projector:


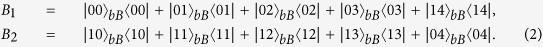


To be precise, the result of bringing in the ancillary systems in state |Ψ〉_*ab*_, and then operating with *B*_1_ on systems *bB*, is that each of the initial states is transformed as:


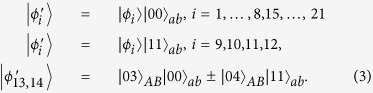


Let us now describe how the parties can proceed from here to distinguish the states. Alice makes a seven-outcome projective measurement, and we begin by considering the first outcome, *A*_1_ = |1〉_*a*_〈1| ⊗ |3 + 4〉_*A*_〈3 + 4|. The only remaining possibility is 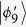, which has thus been successfully identified. In the same way, Alice can identify 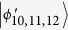 by three projectors *A*_2_ = |1〉_*a*_〈1| ⊗ |3 − 4〉_*A*_〈3 − 4|, *A*_3_ = |1〉_*a*_〈1| ⊗ |1 + 2〉_*A*_〈1 + 2|, *A*_4_ = |1〉_*a*_〈1| ⊗ |1 − 2〉_*A*_〈1 − 2|, respectively.

Using a rank-1 projector *A*_5_ = |0〉_*a*_〈0| ⊗ |4〉_*A*_〈4| onto the Alice’s Hilbert space, it leaves 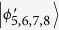 and annihilates other states in (3). Then, Bob can easily distinguish these four remaining states by projectors onto |0 ± 1〉_*B*_ and |2 ± 3〉_*B*_.

Using a rank-2 projector *A*_6_ = |0〉_*a*_〈0| ⊗ |2〉_*A*_〈2| + |0〉_*a*_〈0| ⊗ |3〉_*A*_〈3| onto the Alice’s Hilbert space, it leaves 
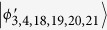
 and annihilates other states in (3). Then, Bob can identify 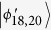 by two projectors *B*_61_ = |0〉_*b*_〈0| ⊗ |1〉_*B*_〈1|, *B*_62_ = |0〉_*b*_〈0| ⊗ |3〉_*B*_〈3|, respectively. When Bob uses a projector *B*_63_ = |0〉_*b*_〈0| ⊗ |0〉_*B*_〈0|, it can leave 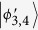 and annihilates other states. Then, Alice can easily distinguish these two remaining states by projectors onto |2 ± 3〉_*A*_. In the same way, we can easily distinguish 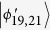.

Alice’s last outcome is a rank-3 projector onto the remaining part of Alice’s Hilbert space *A*_7_ = |0〉_*a*_〈0| ⊗ |0〉_*A*_〈0| + |0〉_*a*_〈0| ⊗ |1〉_*A*_〈1| + |1〉_*a*_〈1| ⊗ |0〉_*A*_〈0|. This leaves 
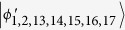
 and annihilates other states. Then, when Bob uses the projector *B*_71_ = |0〉_*b*_〈0| ⊗ |0〉_*B*_〈0|, it leaves 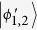, which can be easily distinguished by projectors onto |0 ± 1〉_*A*_. When Bob uses a projector *B*_72_ = |0〉_*b*_〈0| ⊗ (|1〉_*B*_〈1| + |2〉_*B*_〈2|), it can leave 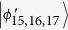 and annihilate other states. Then, Alice can easily identify 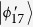 by projector onto |1〉_*A*_, and leave 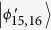 which can be distinguished by Bob. Finally, when Bob uses a projector *B*_73_ = |0〉_*b*_〈0| ⊗ |3〉_*B*_〈3| + |1〉_*b*_〈1| ⊗ |4〉_*B*_〈4|, it leaves the last two states 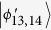 which are orthogonal. In^23^, we know that any two orthogonal states can be distinguished by LOCC. Thus, the two states are locally distinguishable.

For operating with *B*_2_ on systems *bB*, it creates new states which differ from the states (3) only by ancillary systems |00〉_*ab*_ → |11〉_*ab*_ and |11〉_*ab*_ → |00〉_*ab*_. Thus, the latter can be handled using the exact same method as described above for *B*_1_.

That is, we have succeeded in designing a protocol that perfectly distinguishes the states (1) using LOCC with an additional resource of a two-qubit maximally entangled state. This completes the proof.

In addition, a 2 ⊗ 2 maximally entangled state is necessary to distinguish the set of product states for the above protocol. When a partially entangled state, |Ψ〉_*ab*_ = *m*|00〉 + *n*|11〉(*m* ≠ *n*), is shared, the states |*ϕ*_13,14_〉 will be not orthogonal after Bob performs a two-outcome measurement. This means that 1 ebit is necessary for the protocol. Then, it is interesting to design a protocol to distinguish these states with less than one ebit of entanglement, or to prove that there is no any protocol to accomplish it.

### Local distinguishability of orthogonal product states in *m* ⊗ *n*

In this section, we consider the set of locally indistinguishable orthogonal product states in *m* ⊗ *n*^48^, and the structure of these states is also different from ref. 48 only lying in exchange of Alice and Bob. In the following, we separate it into three cases (2*k* + 1) ⊗ (2*l*), (2*k*) ⊗ (2*l*) and (2*k* + 1) ⊗ (2*l* + 1).

### Orthogonal product states in (2*k* + 1) ⊗ (2*l*)

In this subsection, we first present the following locally indistinguishable orthogonal product states in (2*k* + 1) ⊗ (2*l*)^48^. Then, we show that these states can be perfectly distinguished by LOCC with a 2 ⊗ 2 maximally entangled state.


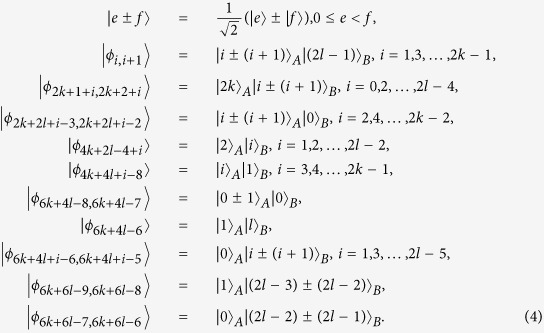


Theorem 2 *In* (2*k* + 1) ⊗ (2*l*), *a* 2 ⊗ 2 *maximally entangled state is sufficient to perfectly distinguish the above 6*(*k* + *l*)*−* *6 states by LOCC*.

*Proof.* Similarly to the proof of Theorem 1, Alice and Bob first share a 2 ⊗ 2 maximally entangled state 
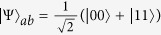
.

Then, Bob performs a two-outcome measurement, each outcome corresponding to a rank-2l projector:


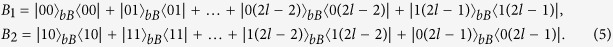


As Theorem 1, we only need to discuss the projector *B*_1_. To be precise, the result of bringing in the ancillary systems in state |Ψ〉_*ab*_, and then operating with *B*_1_ on systems *bB*, is that each of the initial states is transformed as:


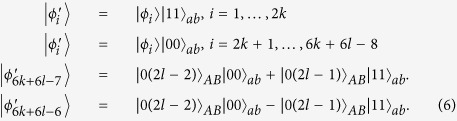


Then, Alice makes a (2k + 3)-outcome projective measurement. Similarly to the proof of Theorem 1, Alice can identify 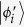, *i* = 1, …, 2*k* by 2*k* projectors *A*_*i*_ = |1〉_*a*_〈1| ⊗ |*i* + (*i* + 1)〉_*A*_〈*i* + (*i* + 1)|, *A*_*i*+1_ = |1〉_*a*_〈1| ⊗ |*i* − (*i* + 1)〉_*A*_〈*i* − (*i* + 1)|, *i* = 1, 3, …, 2*k* − 1, respectively.

Using a rank-1 projector *A*_2*k*+1_ = |0〉_*a*_〈0| ⊗ |2*k*〉_*A*_〈2*k*| onto the Alice’s Hilbert space, it leaves 
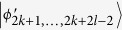
 and annihilates other states in (6). Then, Bob can easily distinguish these remaining states by projectors onto |*i* ± (*i* + 1)〉_*B*_, *i* = 0, 2, …, 2*l* − 4.

When Alice uses a rank-(2k-2) projectors *A*_2*k*+2_ = |0〉_*a*_〈0| ⊗ |2〉_*A*_〈2| + |0〉_*a*_〈0| ⊗ |3〉_*A*_〈3| + … + |0〉_*a*_〈0| ⊗ |2*k* − 1〉_*A*_〈2*k* − 1|, onto the Alice’s Hilbert space, it leaves 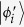, *i* = 2*k* + 2*l* − 1, …, 6*k* + 4*l* − 9 and annihilates other states in (6). Then, Bob can identify 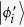, *i* = 4*k* + 2*l* − 2, …, 4*k* + 4*l* − 6 by 2*l* − 3 projectors *B*_(2*k*+2)*i*_ = |0〉_*b*_〈0| ⊗ |*i*〉_*B*_〈*i*|, *i* = 2, …, 2*l* − 2, respectively. When Bob uses projector *B*_(2*k*+2)1_ = |0〉_*b*_〈0| ⊗ |*l*〉_*B*_〈*l*|, it can leave 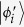, *i* = 4*k* + 2*l* − 3, *i* = 4*k* + 4*l* − 5, …, 6*k* + 4*l* − 9. Then, Alice can easily distinguish these remaining states by projectors onto |*i*〉_*A*_, *i* = 2, 3, …, 2*k* − 1. In the same way, we can easily distinguish 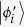, *i* = 2*k* + 2*l* − 1, …, 4*k* + 2*l* − 4.

Alice’s last outcome is a rank-3 projector onto the remaining part of Alice’s Hilbert space *A*_2*k*+3_ = |0〉_*a*_〈0| ⊗ |0〉_*A*_〈0| + |0〉_*a*_〈0| ⊗ |1〉_*A*_〈1| + |1〉_*a*_〈1| ⊗ |0〉_*A*_〈0|. This leaves 
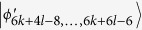
 and annihilates other states. Then, Bob uses the projector *B*_(2*k*+3)1_ = |0〉_*b*_〈0| ⊗ |0〉_*B*_〈0|, which leaves 
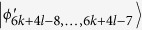
. And these states can be easily distinguished by projectors onto |0 ± 1〉_*A*_. When Bob uses the projector *B*_(2*k*+3)2_ = |0〉_*b*_〈0| ⊗ (|1〉_*B*_〈1| + |2〉_*B*_〈2| + … + |(2*l* − 4)〉_*B*_〈(2*l* − 4)|), it can leave 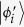, *i* = 6*k* + 4*l* − 6, …, 6*k* + 6*l* − 10. Then, Alice can easily identify 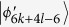 by projector onto |1〉_*A*_, and leave 
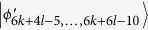
 which can be distinguished by Bob. Finally, when Bob uses the projector *B*_(2*k*+3)3_ = |0〉_*b*_〈0| ⊗ |(2*l* − 3)〉_*B*_〈(2*l* − 3)| + |0〉_*b*_〈0| ⊗ |(2*l* − 2)〉_*B*_〈(2*l* − 2)| + |1〉_*b*_〈1| ⊗ |(2*l* − 1)〉_*B*_〈(2*l* − 1)|, it leaves the last four states 
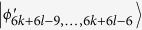
. Then, Alice uses two projectors *A*_31_ = |0〉_*a*_〈0| ⊗ |1〉_*A*_〈1| and *A*_32_ = |0〉_*a*_〈0| ⊗ |0〉_*A*_〈0| + |1〉_*a*_〈1| ⊗ |0〉_*A*_〈0| which leave two sets of orthogonal states 
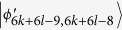
 and 
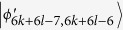
, respectively. According to ref. 23, we know that the two sets of states can also be distinguished by LOCC.

Therefore, a 2 ⊗ 2 maximally entangled state is sufficient to perfectly distinguish the states (4) by LOCC. This completes the proof.

### Orthogonal product states in (2*k*) ⊗ (2*l*)

In this subsection, we consider the following locally indistinguishable orthogonal product states in (2*k*) ⊗ (2*l*)^48^.


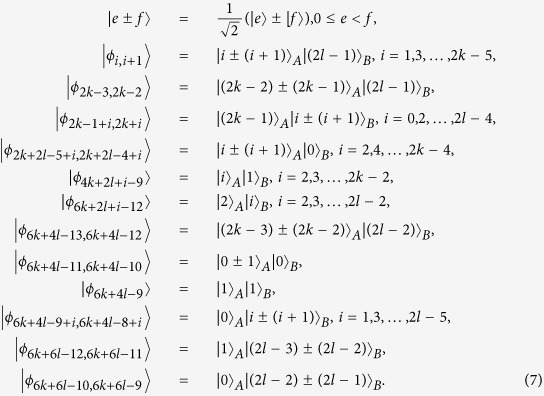


In the following, we present that the above states are LOCC distinguishable with a 2 ⊗ 2 maximally entangled state.

Theorem 3 *In* (*2k*) ⊗ (*2l*), *a* 2 ⊗ 2 *maximally entangled state is sufficient to perfectly distinguish the above 6*(*k* + *l*)−*9 states by LOCC*.

*Proof*. Similarly to the proof of Theorem 2, Alice and Bob first share a 2 ⊗ 2 maximally entangled state 
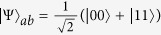
. Then, Bob performs the following measurement:


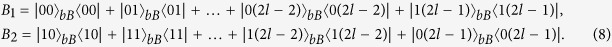


As Theorem 1, we only need to discuss the projector *B*_1_. In the same way, the initial states are transformed as:


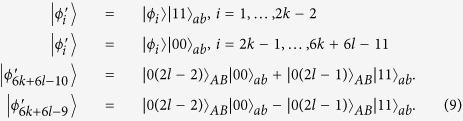


Then, Alice makes a (2k + 1)-outcome projective measurement. Similarly to the proof of Theorem 2, Alice can identify 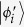, *i* = 1, …, 2*k* − 2 by 2*k* − 2 projectors *A*_*j*_ = |1〉_*a*_〈1| ⊗ |*i* + (*i* + 1)〉_*A*_〈*i* + (*i* + 1)|, *A*_*j*+1_ = |1〉_*a*_〈1| ⊗ |*i* − (*i* + 1)〉_*A*_〈*i* − (*i* + 1)|, *i* = 1, 3, …, 2*k* − 5, *j* = *i*, *i* = 2*k* − 2, *j* = *i* − 1, respectively.

Using a rank-1 projector *A*_2*k*_ − _1_ = |0〉_*a*_〈0| ⊗ |2*k*〉_*A*_〈2*k*| onto the Alice’s Hilbert space, it leaves 
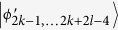
 which can be easily distinguished with Bob by projectors onto |*i* ± (*i* + 1)〉_*B*_, *i* = 0, 2, …, 2*l* − 4.

When Alice uses a rank-(2k-3) projector *A*_2*k*_ = |0〉_*a*_〈0| ⊗ |2〉_*A*_〈2| + |0〉_*a*_〈0| ⊗ |3〉_*A*_〈3| + … + |0〉_*a*_〈0| ⊗ |2*k* − 2〉_*A*_〈2*k* − 2|, it leaves 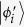, *i* = 2*k* + 2*l* − 3,…, 6*k* + 4*l* − 12. Similarly to the proof of Theorem 2, these states can also be distinguished by LOCC.

For the last rank-3 projector *A*_2*k*+1_ = |0〉_*a*_〈0| ⊗ |0〉_*A*_〈0| + |0〉_*a*_〈0| ⊗ |1〉_*A*_〈1| + |1〉_*a*_〈1| ⊗ |0〉_*A*_〈0|, it leaves 
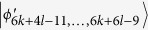
. Then, when Bob uses the projector *B*_(2*k*+1)1_ = |0〉_*b*_〈0| ⊗ |0〉_*B*_〈0|, it leaves 
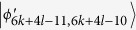
, which can be easily distinguished by projectors onto |0 ± 1〉_*A*_. When Bob uses the projector *B*_(2*k*+1)2_ = |0〉_*b*_〈0| ⊗ (|1〉_*B*_〈1| + |2〉_*B*_〈2| + … + |(2*l* − 4)〉_*B*_〈(2*l* − 4)|), it can leave 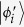, *i* = 6*k* + 4*l* − 9, …, 6*k* + 6*l* − 13. Then, Alice can identify 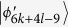 by projector onto |1〉_*A*_, and leave 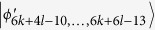 which can be distinguished by Bob. Finally, when Bob uses the projector *B*_(2*k*+1)3_ = |0〉_*b*_〈0| ⊗ |(2*l* − 3)〉_*B*_〈(2*l* − 3)| + |0〉_*b*_〈0| ⊗ |(2*l* − 2)〉_*B*_〈(2*l* − 2)| + |1〉_*b*_〈1| ⊗ |(2*l* − 1)〉_*B*_〈(2*l* − 1)|, it leaves the last four states 
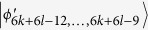
. Similarly to the proof of Theorem 2, these states can also be distinguished by LOCC.

Thus, the states (7) can be perfectly distinguished using LOCC with an additional resource of a 2 ⊗ 2 maximally entangled state. This completes the proof.

### Orthogonal product states in (2*k* + 1) ⊗ (2*l* + 1)

In this subsection, we consider a generalization of (1) to higher-dimensional systems (2*k* + 1) ⊗ (2*l* + 1)^48^. Then, we present that these states can be perfectly distinguished by LOCC with a 2 ⊗ 2 maximally entangled state.


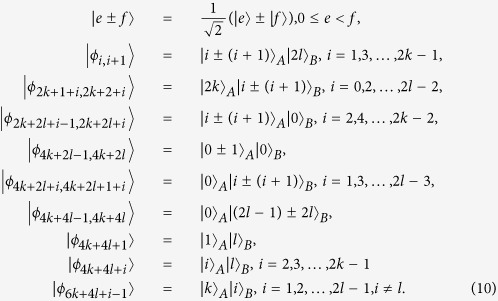


Theorem 4 *In* (2*k* + 1) ⊗ (2*l* + 1), *a* 2 ⊗ 2 *maximally entangled state is sufficient to perfectly distinguish the above 6*(*k* + *l*)−*3 states by LOCC*.

In (2*k* + 1) ⊗ (2*l* + 1), the states (10) are a generalization of the states (1). Thus, the proof is similar to the proof of Theorem 1. In the following, we only give a simple proof.

*Proof*. In the same way, Alice and Bob first share a 2 ⊗ 2 maximally entangled state 
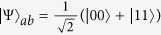
. Then, Bob performs the following measurement: *B*_1_ = |00〉_*bB*_〈00| + |01〉_*bB*_〈01| + … + |0(2*l* − 1)〉_*bB*_〈0(2*l* − 1)| + |1(2*l*)〉_*bB*_〈1(2*l*)| and *B*_2_ = |10〉_*bB*_〈10| + |11〉_*bB*_〈11| + … + |1(2*l* − 1)〉_*bB*_〈1(2*l* − 1)| + |0(2*l*)〉_*bB*_〈0(2*l*)|.

For *B*_1_, the initial states are transformed as:


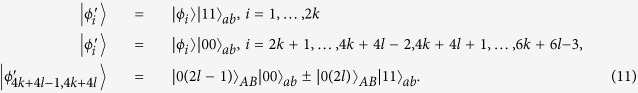


In the same way, it is easily to distinguish 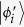, *i* = 1, …, 4*k* + 2*l* − 2, 4*k* + 4*l* + 2, …, 6*k* + 6*l *− 3.

Alice’s last outcome is a rank-3 projector *A*_2*k*+3_ = |0〉_*a*_〈0| ⊗ |0〉_*A*_〈0| + |0〉_*a*_〈0| ⊗ |1〉_*A*_〈1| + |1〉_*a*_〈1| ⊗ |0〉_*A*_〈0|, which leaves 
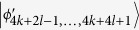
 and annihilates other states. Then, when Bob uses the projector *B*_(2*k*+3)1_ = |0〉_*b*_〈0| ⊗ |0〉_*B*_〈0|, it can leave 
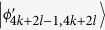
, which can be easily distinguished by projectors onto |0 ± 1〉_*A*_. When Bob uses the projector *B*_(2*k*+3)2_ = |0〉_*b*_〈0| ⊗ (|1〉_*B*_〈1| + |2〉_*B*_〈2| + … + |(2*l* − 2)〉_*B*_〈(2*l* − 2)|), it can leave 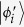, *i* = 4*k* + 2*l* + 1, …, 4*k* + 4*l* − 2, 4*k* + 4*l* + 1. Then, Alice can easily identify 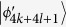 by a projector onto |1〉_*A*_, and leave 
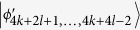
 which can be distinguished by Bob. Finally, Bob uses the projector *B*_(2*k*+3)3_ = |0〉_*b*_〈0| ⊗ |(2*l* − 1)〉_*B*_〈(2*l* − 1)| + |1〉_*b*_〈1| ⊗ |2*l*〉_*B*_〈2*l*|, which leaves the last two orthogonal states 
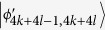
. And the two states can also be distinguished by LOCC^23^.

That is, we have succeeded in designing a protocol to distinguish the states (10) by LOCC with a two-qubit maximally entangled state. This completes the proof.

In the proof, the important idea is that entanglement provides multiple Hilbert space, and that the parties can, independently of one another, act on these subspaces in ways that differ from one subspace to the next. This allows an apart of Hilbert space such that initially nonorthogonal pairs of local states end up being orthogonal, aiding the process of distinguishing the set of states. It should be noted that our protocols do not rely on details of the individual states, but only on the general way they are distributed through the space.

As Theorem 1, a 2 ⊗ 2 maximally entangled state is also necessary to distinguish these product states in *m* ⊗ *n* for the above protocol. Furthermore, it will be good to do the analysis using 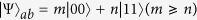 as a resource. In particular, we are interesting in the optimal probability of distinguishing the states using such a state, and whether it is better than 2*n*^2^, which is the optimal probability of converting such a state into a maximally entangled state, where *m*≥*n*. However, we have not succeeded in doing so. Hence, it remains an open question whether it is possible to distinguish these states with less than one ebit of entanglement.

In addition, each of the locally indistinguishable orthogonal product states that we consider can be extended to a complete “nonlocality without entanglement” basis^48^. Therefore, the latter can also be distinguished by the same (or slightly altered) protocols.

## Conclusion

In this paper, we present that the locally indistinguishable orthogonal product states in *m* ⊗ *n*, which are constructed by Wang *et al.*^48^, can be perfectly distinguished by LOCC with a 2 ⊗ 2 maximally entangled state. Our results show that the locally indistinguishable quantum states may become distinguishable with a small amount of entanglement resources. And we hope that these results can lead to a better understanding for the relationship between nonlocality and entanglement. Recently, Bandyopadhyay *et al.*^49^ explore the question of entanglement as a universal resource for implementing quantum measurements by LOCC. And they show that for most multipartite systems (consisting of three or more subsystems), there is no entangled state from the same space that can enable all measurements by LOCC. Thus, it is also interesting to look for entanglement as a resource to locally distinguish multipartite quantum states.

## Additional Information

**How to cite this article**: Zhang, Z.-C. *et al.* Entanglement as a resource to distinguish orthogonal product states. *Sci. Rep.* **6**, 30493; doi: 10.1038/srep30493 (2016).
